# Disease Severity of Respiratory Syncytial Virus Compared with COVID-19 and Influenza Among Hospitalized Adults Aged ≥60 Years — IVY Network, 20 U.S. States, February 2022–May 2023

**DOI:** 10.15585/mmwr.mm7240a2

**Published:** 2023-10-06

**Authors:** Diya Surie, Katharine A. Yuengling, Jennifer DeCuir, Yuwei Zhu, Manjusha Gaglani, Adit A. Ginde, H. Keipp Talbot, Jonathan D. Casey, Nicholas M. Mohr, Shekhar Ghamande, Kevin W. Gibbs, D. Clark Files, David N. Hager, Harith Ali, Matthew E. Prekker, Michelle N. Gong, Amira Mohamed, Nicholas J. Johnson, Jay S. Steingrub, Ithan D. Peltan, Samuel M. Brown, Aleda M. Leis, Akram Khan, Catherine L. Hough, William S. Bender, Abhijit Duggal, Jennifer G. Wilson, Nida Qadir, Steven Y. Chang, Christopher Mallow, Jennie H. Kwon, Matthew C. Exline, Adam S. Lauring, Nathan I. Shapiro, Cristie Columbus, Ivana A. Vaughn, Mayur Ramesh, Basmah Safdar, Natasha Halasa, James D. Chappell, Carlos G. Grijalva, Adrienne Baughman, Todd W. Rice, Kelsey N. Womack, Jin H. Han, Sydney A. Swan, Indrani Mukherjee, Nathaniel M. Lewis, Sascha Ellington, Meredith L. McMorrow, Emily T. Martin, Wesley H. Self, IVY Network

**Affiliations:** ^1^Coronavirus and Other Respiratory Viruses Division, National Center for Immunization and Respiratory Diseases, CDC; ^2^Vanderbilt University Medical Center, Nashville, Tennessee; ^3^Baylor Scott & White Health, Temple, Texas; ^4^Texas A&M University College of Medicine, Temple, Texas; ^5^Baylor, Scott & White Health, Dallas, Texas; ^6^University of Colorado School of Medicine, Aurora, Colorado; ^7^University of Iowa, Iowa City, Iowa; ^8^Wake Forest University Baptist Medical Center, Winston-Salem, North Carolina; ^9^Johns Hopkins Hospital, Baltimore, Maryland; ^10^Hennepin County Medical Center, Minneapolis, Minnesota; ^11^Montefiore Healthcare Center, Albert Einstein College of Medicine, New York, New York; ^12^University of Washington School of Medicine, Seattle, Washington; ^13^Baystate Medical Center, Springfield, Massachusetts; ^14^Intermountain Medical Center and University of Utah, Salt Lake City, Utah; ^15^University of Michigan School of Public Health, Ann Arbor, Michigan; ^16^Oregon Health & Science University Hospital, Portland, Oregon; ^17^Emory University School of Medicine, Atlanta, Georgia; ^18^Cleveland Clinic, Cleveland, Ohio; ^19^Stanford University School of Medicine, Stanford, California; ^20^Ronald Reagan-UCLA Medical Center, Los Angeles, California; ^21^University of Miami, Miami, Florida; ^22^Washington University, St. Louis, Missouri; ^23^The Ohio State University Wexner Medical Center, Columbus, Ohio; ^24^University of Michigan School of Medicine, Ann Arbor, Michigan; ^25^Beth Israel Deaconess Medical Center, Boston, Massachusetts; ^26^Henry Ford Health, Detroit, Michigan; ^27^Yale University School of Medicine, New Haven, Connecticut; ^28^Influenza Division, National Center for Immunization and Respiratory Diseases, CDC.

**Summary**

**What is already known about this topic?**

In June 2023, CDC recommended the first respiratory syncytial virus (RSV) vaccines for adults aged ≥60 years using shared clinical decision-making. Understanding the severity of RSV disease is needed to guide this clinical decision-making.

**What is added by this report?**

During February 2022–May 2023, hospitalizations for RSV were less frequent but were associated with more severe disease than were hospitalizations for COVID-19 or influenza, including receipt of standard flow oxygen therapy, high-flow nasal cannula or noninvasive ventilation, and intensive care unit admission.

**What are the implications for public health practice?**

The potential for severe RSV disease among older adults is important to consider as part of shared clinical decision-making when assessing the benefit of RSV vaccination among adults aged ≥60 years.

## Abstract

On June 21, 2023, CDC’s Advisory Committee on Immunization Practices recommended respiratory syncytial virus (RSV) vaccination for adults aged ≥60 years, offered to individual adults using shared clinical decision-making. Informed use of these vaccines requires an understanding of RSV disease severity. To characterize RSV-associated severity, 5,784 adults aged ≥60 years hospitalized with acute respiratory illness and laboratory-confirmed RSV, SARS-CoV-2, or influenza infection were prospectively enrolled from 25 hospitals in 20 U.S. states during February 1, 2022–May 31, 2023. Multivariable logistic regression was used to compare RSV disease severity with COVID-19 and influenza severity on the basis of the following outcomes: 1) standard flow (<30 L/minute) oxygen therapy, 2) high-flow nasal cannula (HFNC) or noninvasive ventilation (NIV), 3) intensive care unit (ICU) admission, and 4) invasive mechanical ventilation (IMV) or death. Overall, 304 (5.3%) enrolled adults were hospitalized with RSV, 4,734 (81.8%) with COVID-19 and 746 (12.9%) with influenza. Patients hospitalized with RSV were more likely to receive standard flow oxygen, HFNC or NIV, and ICU admission than were those hospitalized with COVID-19 or influenza. Patients hospitalized with RSV were more likely to receive IMV or die compared with patients hospitalized with influenza (adjusted odds ratio = 2.08; 95% CI = 1.33–3.26). Among hospitalized older adults, RSV was less common, but was associated with more severe disease than COVID-19 or influenza. High disease severity in older adults hospitalized with RSV is important to consider in shared clinical decision-making regarding RSV vaccination.

## Introduction

Respiratory syncytial virus (RSV) is increasingly recognized as an important cause of severe respiratory disease in older adults. In the United States, an estimated 60,000–160,000 RSV-associated hospitalizations and 6,000–10,000 RSV-associated deaths occur each year among adults aged ≥65 years (*1*). On June 21, 2023, CDC’s Advisory Committee on Immunization Practices recommended RSV vaccination for adults aged ≥60 years using shared clinical decision-making^†^ (*1*). Understanding the severity of RSV disease compared with that of other respiratory viral diseases in older adults is needed to guide this shared patient-provider clinical decision-making.

## Methods

During February 1, 2022–May 31, 2023, adults aged ≥60 years with acute respiratory illness^§^ and laboratory-confirmed RSV, SARS-CoV-2, or influenza infection who were admitted to any of 25 hospitals in 20 U.S. states participating in the Investigating Respiratory Viruses in the Acutely Ill (IVY) Network^¶^ were eligible for inclusion in this analysis. Demographic and clinical data were obtained from patient or proxy interview and medical records, including in-hospital outcomes observed by day 28 of hospitalization. Upper respiratory specimens were collected from enrolled patients near the time of admission and tested at a central laboratory (Vanderbilt University Medical Center, Nashville, Tennessee) by reverse transcription–polymerase chain reaction for RSV, SARS-CoV-2, and influenza. Patients who received a positive RSV, SARS-CoV-2 or influenza result based on either hospital or central laboratory testing within 10 days of illness onset or within 3 days of hospital admission were included.

Severity of RSV disease was compared with COVID-19 and influenza severity using the following in-hospital outcomes: 1) standard flow oxygen therapy, defined as receipt of supplemental oxygen at <30 L/minute; 2) receipt of high-flow nasal cannula (HFNC) or noninvasive ventilation (NIV); 3) intensive care unit (ICU) admission; and 4) receipt of invasive mechanical ventilation (IMV) or death. For this analysis, enrolled patients were excluded if they had confirmed or inconclusive laboratory test results indicating coinfection with RSV, SARS-CoV-2, or influenza or if data for in-hospital outcomes were missing.

In-hospital outcomes were compared among patients hospitalized with RSV disease, COVID-19, and influenza using multivariable logistic regression. Models were adjusted for age, sex, self-reported race and Hispanic or Latino (Hispanic) ethnicity, number of organ systems associated with a chronic medical condition, and U.S. Department of Health and Human Services geographic region. Differences among respiratory viruses were assessed for each outcome; p-values <0.05 were considered statistically significant. All analyses were conducted using SAS software (version 9.4; SAS Institute). This activity was reviewed by CDC, deemed not research, and was conducted consistent with applicable federal law and CDC policy.**

## Results

During February 1, 2022–May 31, 2023, a total of 6,061 adults aged ≥60 years were enrolled in IVY Network with acute respiratory illness and laboratory-confirmed infection with RSV, SARS-CoV-2, or influenza. After exclusion of 277 patients,^††^ 5,784 were included in this analysis, among whom 304 (5.3%) were hospitalized with RSV, 4,734 (81.8%) with COVID-19, and 746 (12.9%) with influenza. Substantial seasonal variation in hospital admissions was observed for RSV and influenza, but SARS-CoV-2 admissions exhibited less seasonal variation (Figure).

**FIGURE:**
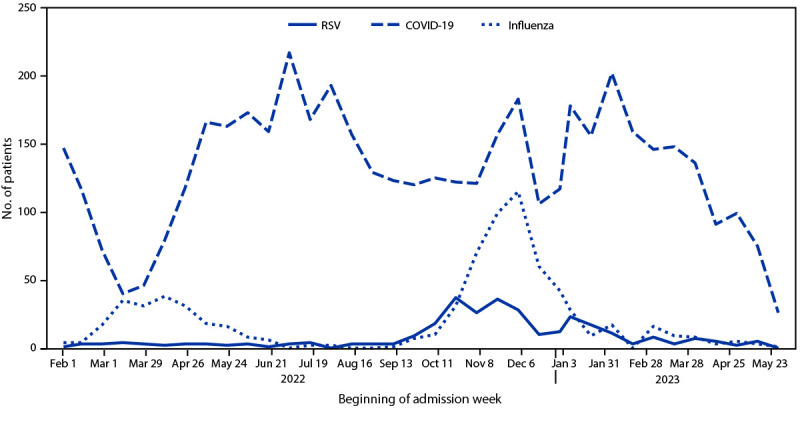
Date of admission for adults aged ≥60 years hospitalized with respiratory syncytial virus, COVID-19, or influenza — Investigating Respiratory Viruses in the Acutely Ill Network, 25 hospitals, 20 U.S. states,* February 1, 2022–May 31, 2023 **Abbreviation:** RSV = respiratory syncytial virus. * https://www.cdc.gov/flu/vaccines-work/ivy.htm

The median age of adults hospitalized with RSV (72 years) was similar to the age of those hospitalized with COVID-19 (74 years) and influenza (71 years) (Table 1). Among patients hospitalized with RSV or COVID-19, percentages of non-Hispanic Black or African American (Black) patients were similar (18.1% and 16.8%, respectively); however, among patients hospitalized with influenza, the percentage of Black patients was higher (188; 25.2%). Patients hospitalized with RSV had chronic medical conditions associated with a median of two organ systems, a finding similar to that for patients hospitalized with COVID-19 or influenza. Among the 5,784 included patients, 4,713 (81.5%) had received ≥1 dose of original (ancestral) monovalent or bivalent (ancestral and BA.4/5) COVID-19 vaccine, and 2,795 (48.3%) had received seasonal influenza vaccination.^§§^

**TABLE 1:** Characteristics of adults aged ≥60 years hospitalized with respiratory syncytial virus, COVID-19, or influenza — Investigating Respiratory Viruses in the Acutely Ill Network, 25 hospitals,* 20 U.S. states, February 1, 2022–May 31, 2023

Characteristic	No. (%)			
	Total N = 5,784	RSV n = 304	COVID-19 n = 4,734	Influenza n = 746
**Age, yrs, median (IQR)**	**74 (67–81)**	72 (66–80)	74 (67–82)	71 (65–79)
**Age group, yrs**				
60–69	**2,038 (35.2)**	116 (38.2)	1,601 (33.8)	321 (43.0)
70–79	**1,978 (34.2)**	110 (36.2)	1,623 (34.3)	245 (32.8)
≥80	**1,768 (30.6)**	78 (25.7)	1,510 (31.9)	180 (24.1)
**Race and ethnicity**				
Black or African American, non-Hispanic	**1,038 (17.9)**	55 (18.1)	795 (16.8)	188 (25.2)
White, non-Hispanic	**3,659 (63.3)**	178 (58.6)	3,095 (65.4)	386 (51.7)
Hispanic or Latino, any race	**702 (12.1)**	44 (14.5)	543 (11.5)	115 (15.4)
Other race, non-Hispanic^†^	**293 (5.1)**	22 (7.2)	224 (4.7)	47 (6.3)
Other^§^	**92 (1.6)**	5 (1.6)	77 (1.6)	10 (1.3)
**Sex**				
Female	**2,898 (50.1)**	173 (56.9)	2,326 (49.1)	399 (53.5)
Male	**2,886 (49.9)**	131 (43.1)	2,408 (50.9)	347 (46.5)
HHS region*				
1	**1,117 (19.3)**	41 (13.5)	971 (20.5)	105 (14.1)
2	**337 (5.8)**	27 (8.9)	239 (5.0)	71 (9.5)
3	**221 (3.8)**	8 (2.6)	199 (4.2)	14 (1.9)
4	**998 (17.3)**	59 (19.4)	812 (17.2)	127 (17.0)
5	**881 (15.2)**	37 (12.2)	712 (15.0)	132 (17.7)
6	**676 (11.7)**	25 (8.2)	550 (11.6)	101 (13.5)
7	**328 (5.7)**	29 (9.5)	246 (5.2)	53 (7.1)
8	**731 (12.6)**	51 (16.8)	574 (12.1)	106 (14.2)
9	**295 (5.1)**	19 (6.3)	257 (5.4)	19 (2.6)
10	**200 (3.5)**	8 (2.6)	174 (3.7)	18 (2.4)
**No. of organ systems with a chronic medical condition, median (IQR)^¶^**	**2 (2–3)**	2 (2–3)	2 (2–4)	2 (2–3)
COVID-19 vaccination status**				
Unvaccinated	**997 (17.2)**	29 (9.5)	837 (17.7)	131 (17.6)
Vaccinated^††^	**4,713 (81.5)**	274 (90.1)	3,834 (81.0)	605 (81.1)
**Influenza vaccination status^§§^**				
Unvaccinated	**2,548 (44.1)**	131 (43.1)	2,026 (42.8)	391 (52.4)
Vaccinated^¶¶^	**2,795 (48.3)**	147 (48.4)	2,343 (49.5)	305 (40.9)

**Abbreviations**: HHS = U.S. Department of Health and Human Services; RSV = respiratory syncytial virus.

* Hospitals by HHS region include *Region 1:* Baystate Medical Center (Springfield, Massachusetts), Beth Israel Deaconess Medical Center (Boston, Massachusetts), and Yale University (New Haven, Connecticut); *Region 2:* Montefiore Medical Center (New York, New York); *Region 3:* Johns Hopkins Hospital (Baltimore, Maryland); *Region 4:* Emory University Medical Center (Atlanta, Georgia), University of Miami Medical Center (Miami, Florida), Vanderbilt University Medical Center (Nashville, Tennessee), and Wake Forest University Baptist Medical Center (Winston-Salem, North Carolina); *Region 5:* Cleveland Clinic (Cleveland, Ohio), Hennepin County Medical Center (Minneapolis, Minnesota), Henry Ford Health (Detroit, Michigan); The Ohio State University Wexner Medical Center (Columbus, Ohio), and University of Michigan Hospital (Ann Arbor, Michigan); *Region 6:* Baylor Scott & White Medical Center (Temple, Texas) and Baylor University Medical Center (Dallas, Texas); *Region 7:* Barnes-Jewish Hospital (St. Louis, Missouri) and University of Iowa Hospitals (Iowa City, Iowa); *Region 8:* Intermountain Medical Center (Murray, Utah) and UCHealth, University of Colorado Hospital (Aurora, Colorado); *Region 9:* University of Arizona Medical Center (Phoenix, Arizona), Stanford University Medical Center (Stanford, California), and UCLA Medical Center (Los Angeles, California); and *Region 10:* Oregon Health & Science University Hospital (Portland, Oregon) and University of Washington (Seattle, Washington).

^†^ Other race, non-Hispanic includes American Indian or Alaska Native, Asian, and Native Hawaiian or other Pacific Islander, which were combined because of small counts.

^§^ Other includes patients who self-reported their race and ethnicity as “other” and those for whom race and ethnicity were unknown.

**^¶^** Organ systems with chronic medical conditions include cardiovascular disease, neurologic disease, pulmonary disease, gastrointestinal disease, endocrine disease, kidney disease, hematologic disease, autoimmune disease, and immunocompromising conditions.

** A total of 74 (1.3%) patients were missing COVID-19 vaccination status, including one (0.3%) among RSV patients, 63 (1.3%) among COVID-19 patients, and 10 (1.3) among influenza patients.

^††^ Includes patients with receipt of ≥1 dose of original (ancestral) monovalent vaccines, specifically BNT1262b2, (Pfizer-BioNTech), mRNA-1273 (Moderna), NVX-CoV2373 (Novavax), Ad26.COV2.S (Janssen [Johnson & Johnson]) or patients with receipt of ≥1 dose bivalent (ancestral and BA.4/5) vaccines, specifically BNT1262b2 bivalent vaccine (Pfizer-BioNTech) and mRNA-1273.222 (Moderna) bivalent vaccine. Patients who received bivalent vaccination might have previously received 1–6 doses of the original (ancestral) monovalent vaccines.

^§§^ A total of 441 (7.6%) patients were missing influenza vaccination status, including 26 (8.6%) RSV patients, 365 (7.7%) COVID-19 patients, and 50 (6.7%) influenza patients.

^¶¶^ Patients were classified as vaccinated against influenza if they received season-specific influenza vaccination based on the period in which they were enrolled.

In adjusted analyses comparing RSV severity with COVID-19, patients hospitalized with RSV were more likely than hospitalized COVID-19 patients or hospitalized influenza patients were to receive standard flow oxygen (adjusted odds ratio [aOR] = 2.97 [COVID-19] and 2.07 [influenza]), HFNC or NIV (aOR = 2.25 [COVID-19] and 1.99 [influenza]), or to be admitted to an ICU (aOR = 1.49 [COVID-19] and 1.55 [influenza]) (Table 2). The odds of the composite outcome of IMV or death between patients hospitalized with RSV and patients hospitalized with COVID-19 was similar (aOR 1.39; 95% CI = 0.98–1.96); however, among hospitalized adults aged ≥60 years with RSV, the odds of IMV or death were significantly higher compared with hospitalized influenza patients (aOR 2.08; 95% CI = 1.33–3.26).

**TABLE 2:** In-hospital outcomes among adults aged ≥60 years hospitalized with respiratory syncytial virus, COVID-19, or influenza — Investigating Respiratory Viruses in the Acutely Ill Network, 25 hospitals,* 20 U.S. states, February 1, 2022–May 31, 2023

In-hospital outcomes	No./Total no. (%)			RSV vs. COVID-19 aOR^†^ (95% CI)	p-value	RSV vs. influenza aOR^†^ (95% CI)	p-value
	RSV patients n = 304	COVID-19 patients n = 4734	Influenza patients n = 746				
Standard flow oxygen therapy^§^	157/197 (79.7)	2,169/3,726 (58.2)	390/593 (65.8)	2.97 (2.07–4.27)	<0.001	2.07 (1.37–3.11)	<0.001
HFNC or NIV^¶^	59/256 (23.0)	495/4,223 (11.7)	94/687 (13.7)	2.25 (1.65–3.07)	<0.001	1.99 (1.36–2.90)	<0.001
ICU admission	74/304 (24.3)	819/4,734 (17.3)	125/746 (16.8)	1.49 (1.13–1.97)	0.005	1.55 (1.11–2.19)	0.01
IMV or death	41/304 (13.5)	481/4,734 (10.2)	52/746 (7.0)	1.39 (0.98–1.96)	0.07	2.08 (1.33–3.26)	0.001

**Abbreviations**: aOR = adjusted odds ratio; HFNC = high-flow nasal cannula; ICU = intensive care unit; IMV = invasive mechanical ventilation; NIV = noninvasive ventilation; RSV = respiratory syncytial virus.

* https://www.cdc.gov/flu/vaccines-work/ivy.htm

**^†^** Multivariable logistic regression models were adjusted for age, sex, race and ethnicity, number of organ systems with chronic medical conditions, and U.S. Department of Health and Human Services region.

^§^ Standard flow oxygen therapy was defined as receipt of supplemental oxygen therapy at a flow rate <30 L/minute as the highest level of oxygen support received during hospitalization.

^¶^ HFNC or NIV was defined as patients who received either HFNC (oxygen therapy at a flow rate ≥30 L/minute) or NIV as the highest level of oxygen support received during hospitalization.

## Discussion

The findings from this study demonstrate that RSV is an important cause of respiratory virus–associated morbidity and mortality in older adults. In this prospective, multicenter analysis in which all enrolled older adults hospitalized in 20 U.S. states during 2022–2023 received testing for RSV, SARS-CoV-2, and influenza, RSV-associated hospitalizations were less frequent than were COVID-19–associated and influenza-associated hospitalizations; however, clinical outcomes in patients hospitalized with RSV were worse than those among patients hospitalized with COVID-19 or influenza. Because RSV disease is less common than COVID-19 or influenza disease among hospitalized patients, clinicians might be less aware of RSV as a serious respiratory pathogen in older adults.

The findings in this analysis are consistent with those from earlier studies that compared RSV disease severity among hospitalized adults with influenza disease (*2*–*4*). Although outcome definitions vary across studies, most demonstrate that patients hospitalized with RSV disease are more likely to be treated with supplemental oxygen, mechanical ventilation, or ICU admission than are patients hospitalized with influenza disease (*2*–*4*).

An important finding in this analysis is that older adults hospitalized with RSV were also more likely to receive standard flow oxygen therapy, HFNC or NIV, or be admitted to an ICU, compared with patients hospitalized with COVID-19. Few studies have compared RSV severity with that associated with COVID-19, and those that have were completed in 2020, before emergence of the Omicron variant and introduction of COVID-19 vaccines (*4*,*5*). Those studies demonstrated that patients hospitalized with RSV were less likely to experience ICU admission, mechanical ventilation, and in-hospital death than were patients hospitalized with COVID-19. Higher RSV severity relative to that of COVID-19 observed in this analysis is likely due to a combination of factors, including 1) reduced severity of Omicron variant sublineages circulating during the period of this analysis, 2) substantial increases in vaccine- and infection-conferred immunity against SARS-CoV-2, and 3) increases in use of antiviral treatments (*6*,*7*).

The high RSV disease severity observed among older adults in this analysis is important to guide decision-making for RSV vaccination in this population. Although neither of the two clinical trials that led to Food and Drug Administration (FDA) approval of RSV vaccines for older adults was powered to assess protection of RSV vaccination against hospitalization in adults aged ≥60 years, both trials showed moderate to high efficacy of RSV vaccination against lower respiratory tract disease, which is in the causal pathway leading to severe disease (*8*,*9*). Although additional studies are needed to assess protection of these vaccines against severe respiratory disease in older adults, RSV vaccination has the potential to prevent severe respiratory disease and is currently the only approved prevention product available for older adults. 

### Limitations

The findings in this report are subject to at least three limitations. First, it is possible that RSV was preferentially detected among more severely ill patients who were more likely to receive clinical testing for RSV at participating hospitals and be subsequently enrolled. However, all patients with acute respiratory illness who were enrolled in IVY Network also received central testing for RSV, SARS-CoV-2, and influenza. During the period of this analysis, IVY Network enrolled 5,955 patients aged ≥60 years with acute respiratory illness who did not have a clinical diagnosis of RSV, SARS-CoV-2, or influenza, and only 25 (0.4%) received a positive RSV test result, based on central testing. Thus, any potential selection bias related to increased detection of RSV among more severely ill patients is likely minimal. In addition, the consistency of RSV severity findings in this analysis compared with findings from other studies that have used different methods lessens these concerns (*2*,*3*). Second, although COVID-19 and influenza vaccination, as well as antiviral or immunomodulatory treatments, have been shown to reduce severity of in-hospital outcomes, results were presented as unstratified respiratory virus groups to represent the overall population hospitalized with RSV, COVID-19, or influenza during the analysis period. Finally, although sample size was sufficient for the results presented, a larger sample size would have allowed for evaluation of mortality as an independent outcome or adjustment for additional patient characteristics (e.g., immunocompromising conditions).

### Implications for Public Health Practice

These findings suggest that although RSV hospitalizations occur less frequently than COVID-19 or influenza hospitalizations, RSV disease among hospitalized adults aged ≥60 years in the United States during February 2022–May 2023 was more severe than that associated with COVID-19 and influenza. New FDA-approved RSV vaccines for adults aged ≥60 years are expected to prevent lower respiratory tract disease (*1*). Health care providers and older adults should consider RSV disease severity when making a shared clinical decision about RSV vaccination (*1*).

## Footnotes

* These authors contributed equally to this report.

† Unlike age- and risk-based recommendations, for which the default decision should be to vaccinate the patient unless vaccination is contraindicated, shared clinical decision-making recommendations have no default. The decision whether to vaccinate may take into account the best available evidence regarding who would benefit from vaccination; the individual patient’s characteristics, values, and preferences; the vaccine characteristics; and the clinician’s discretion. https://www.cdc.gov/vaccines/acip/acip-scdm-faqs.html

§ Acute respiratory illness was defined as one including any of the following signs and symptoms: fever, cough, shortness of breath, new or worsening findings on chest imaging consistent with pneumonia, or hypoxemia (defined as SpO2 <92% on room air or supplemental oxygen to maintain SpO2 ≥92%). For patients receiving chronic oxygen therapy, hypoxemia was defined as SpO2 below baseline or an escalation in supplemental oxygen use to maintain a baseline SpO2.

¶
https://www.cdc.gov/flu/vaccines-work/ivy.htm

** 45 C.F.R. part 46.102(l)(2), 21 C.F.R. part 56; 42 U.S.C. Sect. 241(d); 5 U.S.C. Sect. 552a; 44 U.S.C. Sect. 3501 et seq.

†† A total of 120 patients were excluded because of laboratory-confirmed coinfections, 226 patients were excluded because of inconclusive laboratory test results, preventing confirmation of coinfections, and five patients were excluded because of missing in-hospital clinical outcomes, yielding 277 total exclusions. These 277 exclusions were not mutually exclusive.

§§ Patients were classified as having been vaccinated against COVID-19 based on receipt of ≥1 dose of original (ancestral) monovalent vaccines (BNT1262b2, [Pfizer-BioNTech], mRNA-1273 [Moderna], NVX-CoV2373 [Novavax], or Ad26.COV2.S [Janssen (Johnson & Johnson)]) or receipt of ≥1 dose of bivalent (ancestral and BA.4/5) vaccine (BNT1262b2 bivalent vaccine [Pfizer-BioNTech] or mRNA-1273.222 [Moderna] bivalent vaccine). Patients who received bivalent vaccine might have previously received 1–6 doses of the original (ancestral) monovalent vaccines. Patients were classified as having been vaccinated against influenza if they had received season-specific influenza vaccination based on the period during which they were enrolled.
